# A New Method of Testing the Functions of the Digestive Apparatus

**Published:** 1908-03

**Authors:** 


### ABSTRACT.

---

### A New Method of Testing the Functions of the Digestive Apparatus.

EINHORN (*Therapeutic Gazette,* January, 1908,) submits a method for investigating the functions of the intestinal tract, the principle of which is the administration of test substances with the food and observation of the effects of the digestive fluids upon these substances.

Practically this test is made as follows: patients are given in a gelatin capsule a string of beads with the following substances attached thereto: catgut, fishbone, meat, thymus, potato, mutton fat. After administering the capsule, every stool is examined with the stool sieve until the bead-string has been recovered. If diarrhea is present the sifting may not be necessary, as the bead-string can readily be seen, usually at the bottom of a glass vessel.

Under normal conditions the bead-string appears after one or two days. It is then rinsed in cold water and examined. If digestion is normal we find that catgut, meat, and potato (except the skin) disappear entirely, thymus and fat almost entirely, whereas the fishbone usually disappears, but occasionally it may be present. The nuclei of the thymus always disappear. In pathological conditions deviations from the normal are observed, not only in regard to the time of recovery of the beads (disturbances of motility), but also in regard to the presence of the food substances (disturbances of the digestive function).

The author divides his cases of intestinal digestive disturbance into two groups:

1. Those of pure nervous intestinal dyspepsia. 2. Those of genuine intestinal dyspepsia.

In that great class of cases of intestinal dyspepsia, in which the starch digestion alone is disturbed, taka-diastase (takamine) has proved of especial value.

---

Brown—There are plenty of books telling how to save life while waiting for the doctor.

Smith—Yes. What we need is one telling the young doctor how to save his life while waiting for the patient.—*London Opinion.*

